# PD-L1-Targeting Nanoparticles for the Treatment of Triple-Negative Breast Cancer: A Preclinical Model

**DOI:** 10.3390/ijms26073295

**Published:** 2025-04-02

**Authors:** Wendy K. Nevala, Liyi Geng, Hui Xie, Noah A. Stueven, Svetomir N. Markovic

**Affiliations:** 1 Mayo Clinic, 200 1st St SW, Rochester, MN 55905, USA; nevala.wendy@mayo.edu (W.K.N.); geng.liyi@mayo.edu (L.G.); stueven.noah@mayo.edu (N.A.S.); 2 Vivasor, 9380 Judicial Dr., San Diego, CA 92121, USA; hxie@vivasor.com

**Keywords:** programmed death ligand 1 (PD-L1), nano-immune conjugate (NIC), triple-negative breast cancer (TNBC), mouse xenograft model

## Abstract

Triple-negative breast cancer (TNBC) is a highly aggressive form of breast cancer. Common treatments following surgical resection include PD-1-targeting checkpoint inhibitors (pembrolizumab), as 20% of tumors are PD-L1 positive with or without systemic chemotherapy. Over the last several years, our laboratory has developed nano-immune conjugates (NIC) in which hydrophobic chemotherapy drugs like paclitaxel (PTX) and SN38, the active metabolite of irinotecan, are made water soluble by formulating them into albumin-based nanoparticles (nab) that are hydrophobically linked to various IgG1 monoclonal antibodies, creating an antigen-targetable nano-immune conjugate. To date, we have successfully tested PTX containing NICs linked to either VEGF- or CD20-targeted antibodies in two phase I clinical trials against multiple relapsed ovarian/uterine cancer or non-Hodgkin’s lymphoma, respectively. Herein, we describe a novel NIC created with either PTX or SN38 that is coated with anti-PD-L1-targeting antibodies for the treatment of a preclinical model of TNBC. In vitro testing suggests that the chemotherapy drug and antibody retain their toxicity and ligand binding capability in the context of the NIC. Furthermore, both the PTX and SN-38 NIC demonstrate superior anti-tumor efficacy relative to antibody and chemotherapy drugs alone in a PD-L1 + MDA-MB-231 human TNBC xenograft model, which could translate clinically to patients with TNBC.

## 1. Introduction

Breast cancer is the most common cancer among women and accounts for 25% of all cancers [1]. Triple-negative breast cancer (TNBC) is the most aggressive form of breast cancer and accounts for 15–20% of breast cancer diagnoses [2]. TNBC is characterized by the lack of estrogen and progesterone receptors, and the human epidermal growth factor receptor 2 [3] and, therefore, these patients do not qualify for receptor-targeted therapy. Furthermore, TNBC is the most likely of all breast cancers to recur following primary therapy, and the prognosis is poor [4,5]. The 5-year survival rate for TNBC is 65% and only 12% if the tumor has metastasized to other tissues [3]. Despite recent advances, new or alternative treatments are needed for TNBC to improve outcomes in this patient population.

Cytotoxic chemotherapy continues to be the cornerstone for the drug therapy of most advanced solid tumors. Standard chemotherapy can elicit strong tumor responses; unfortunately, systemic chemotherapy is associated with significant toxicities that often require dose reductions and even discontinuation of treatment [6,7,8]. Therefore, the development of novel drug formulations of chemotherapy to increase tumor uptake of drugs while limiting off-target toxicity is an area of intense research. These drug formulations include antibody–drug conjugates (ADC) [9,10], encapsulation of hydrophobic drugs in micelles [11,12,13,14,15,16], liposomes [17,18,19,20,21], polymer conjugates [22,23], and nanoparticles [24,25,26,27]. PTX and irinotecan, a mitotic inhibitor and a topoisomerase I inhibitor, respectively, are two chemotherapy drugs that have been the subject of new formulation research [21,25,27]. PTX (Table 1), irinotecan, and its active metabolite, SN-38 (Table 2), have a variety of macromolecular formulations that are in clinical development.

In addition to systemic chemotherapy, the use of immune checkpoint inhibitor (ICI) therapy for the treatment of various cancers has become increasingly popular. For example, antibodies to the programmed death ligand-1 (PD-L1) are FDA-approved in gastric, colorectal, renal, and triple-negative breast cancer, melanoma, and NSCLC [28,29,30]. More recently, ICI has been used in combination with macromolecular formulations of chemotherapy to further improve patient outcomes [31,32,33,34]. The most notable formulation of PTX is nab-PTX (Abraxane), which is FDA-approved for the treatment of advanced pancreatic cancer [35], breast cancer [36], and NSCLC [37]. Specifically, nab-PTX plus anti-PD-L1, atezolizumab, is approved for the treatment of TNBC [33]. Irinotecan has mostly been used to treat colorectal cancer, but recently, IMMU-132, an ADC comprised of SN-38 conjugated to an anti-TROP 2 antibody, has been approved for relapsed and refractory TNBC [9].

PTX and SN-38 are two chemotherapy drugs that are hydrophobic, which can be made hydrophilic by emulsifying them into albumin nanoparticles under high pressure and making the chemotherapy albumin complexes water soluble (nano-albumin, nab). The advantage of these hydrophilic formulations is that they can be administered over a shorter time period with faster distribution into the tissue [38] while significantly reducing injection site toxicity [37]. Over the last several years, our laboratory has spent considerable time developing antibody–drug conjugates that consist of hydrophobic chemotherapeutic agents, including PTX and SN-38, that are packaged within albumin nanoparticles [39,40] and coated with tumor-directed human IgG1 antibodies, creating a nano-immune conjugate (NIC). When these albumin nanoparticles are formed, binding sites on albumin are unmasked that bind clinically relevant, therapeutic antibodies that allow drug targeting of the NIC to the tumor microenvironment. To date, two of our NIC molecules containing PTX nanoparticles (modified nab-PTX) coated with bevacizumab (anti-VEGF) and a PTX-containing nanoparticle coated with rituximab (anti-CD20) have completed phase I clinical testing in heavily pretreated patients with relapsed/refractory metastatic gynecological malignancies and non-Hodgkin’s lymphoma. The results demonstrate that the NICs are safe and exhibit promising objective response (RECIST) rates of 53% [41] and 82% (abstract accepted to ASH December 2023) in patients with metastatic ovarian or uterine cancer or non-Hodgkin’s lymphoma, respectively. Here, we discuss preclinical testing of two anti-PD-L1 antibodies, atezolizumab and STI-3031, targeted NICs containing PTX or SN-38, the active metabolite of irinotecan. We have shown that we can reproducibly manufacture nanoparticles, which in turn can bind to atezolizumab STI-3031 in vitro. The NIC demonstrates higher zeta potential than the nanoparticles alone. We also show that the antibody and chemotherapy drug retain their function in the context of the NIC, with similar toxicity and ligand binding as chemotherapy and antibody drugs alone. Furthermore, in vivo testing in a xenograft model of TNBC shows enhanced tumor activity and survival in mice treated with the PD-L1-targeted NIC to both drugs given in combination, suggesting effective PD-L1 targeting.

**Table 1:** PTX macromolecule and nanoparticle formulations in clinical testing.

Drug	Formulation	Disease	Phase	Reference
PNU166945	Polymer conjugate of PTX prodrug	Solid Tumors	Phase I	[22]
EndoTAG-1	PTX in neutral and cationic liposomes	Advanced Head and Neck Cancer and Triple-Negative Breast Cancer	Phase I/II	[17,20]
Ang1005	PTX covalently linked to Angiopep-2 peptide	Recurrent CNS metastasis from breast cancer	Phase II	[42]
OncoGel	PTX in biodegradable Gel	Esophageal Cancer	Phase II	[43]
Genexol-PM	Polymeric micelle encapsulation of PTX	Metastatic Breast Cancer, NSCLC, Ovarian Cancer	Phase II	[11,14,15,16]
NK105	Polymeric micelle encapsulation of PTX	Recurrent Breast Cancer	Phase III	[12]
CT-1203	Macromolecule PTX conjugate with polyglutamic acid	NSCLC	Phase III	[44]
Abraxane	Albumin-bound PTX nanoparticle	NSCLC, Breast Cancer, advanced Pancreatic Cancer	Approved	[10,45,46]

**Table 2:** Irinotecan and SN-38 macromolecule and nanoparticle formulations in clinical testing.

Drug	Formulation	Disease	Phase	Reference
LE-SN38	Liposome-encapsulated SN-38	Colorectal Cancer after progression on oxaliplatin	Preclinical Phase I	[18,19]
IMMU-130	Antibody–drug conjugate of SN-38 and anti-CEACAM5	Colorectal Cancer	Phase I/II	[24]
NK012	Polymeric micelle encapsulated SN-38	Colorectal Cancer	Phase II	[13]
IMMU-132	Antibody–drug conjugate of SN-38 and anti-TROP2	Recurrent and refractory Triple-Negative Breast Cancer	Approved	[9]
Onivyde	Irinotecan liposome	Colorectal Cancer, Pancreatic Cancer, Esophago-gastric Cancer	Approved	[21]

## 2. Results

**Figure 1:**
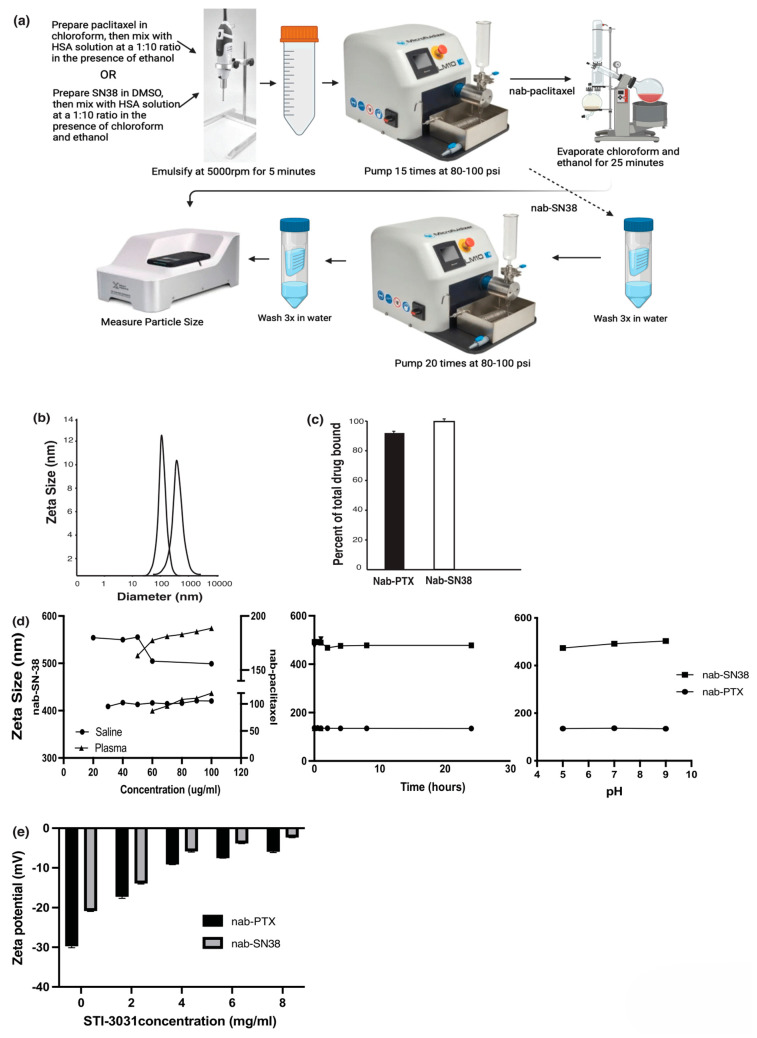
Nanoparticle manufacturing schematic and physiochemical properties of nanoparticles and NIC formation; (**a**) is a schematic representation of the manufacturing process for nab-PTX and nab-SN38 made in Biorender (**b**) Nanoparticles were diluted to 100 µg/mL, and their size was determined with a zeta-sizer, and sizes are represented in nm. (**c**) HPLC was performed to measure the total amount of drug that was bound to the nanoparticles and 90–100% of the 10 mg/mL bound to albumin. (**d**) Nanoparticles were diluted in saline or simulated plasma from 20 to 100 µg/mL, and the size of the particles was determined with the zeta-sizer (left panel); nanoparticles were diluted in saline at 50 µg/mL, and their stability was determined over time (middle panel) and pH (right panel). (**e**) STI-3031 was incubated with the nanoparticles in increasing concentration (0–8 mg/mL), and zeta potential was determined with a zeta-sizer.

Nanoparticle manufacturing and testing: Over the last several years, we have developed a reproducible nanoparticle production process in which highly hydrophobic cytotoxic drugs are bound to albumin to form nanoparticles (Figure 1a) from 130 to 400 nM (Figure 1b), depending on the drug utilized. By doing this, the hydrophobic cytotoxic agent is made water soluble. Once the nanoparticles are manufactured, HPLC is utilized to measure the amount of drug that is bound to the albumin nanoparticles, 91% for PTX and 99% for SN38 (Figure 1c).

To determine stability, the nab-PTX and nab-SN38 were diluted in saline and simulated plasma (5% human serum albumin in HPLC grade water) at drug concentrations ranging from 20 to 100 µg/mL, and nanoparticle size was measured by a zeta-sizer (Malvern Instruments, Malvern, UK). The PTX nanoparticles were stable at 30 µg/mL and 60 µg/mL in saline and simulated plasma, respectively (Figure 1d, right panel). Nab-SN38 nanoparticles were more stable than nab-PTX with particles detected at 20 µg/mL and 50 µg/mL in saline and simulated plasma, respectively (Figure 1d, right panel). These are the lowest concentrations in which intact nanoparticles can be detected by the zeta-sizer. In addition, nab-PTX and nab-SN38 were diluted to 50 µg/mL in saline, and stability was determined from 0 to 24 h (Figure 1d, middle panel) and at pH 5.0, 7.0, and 9.0 (Figure 1d, right panel). The two drug nanoparticles were found to be stable at all time points and pH tested. Also, at time points 2, 4, 8, and 24 h, the nanoparticle supernatant was tested for free chemotherapy drug, and no free drug was detected, suggesting that the drug is not released from the albumin particles. Instead, the larger macromolecules dissociate into functional trimers, including an albumin, PTX, and antibody molecule. Similar results were seen with bevacizumab and rituximab nab-PTX particles [39,40].

After the nanoparticles have been manufactured and lyophilized, nano-immune conjugates (NIC) are created by co-incubating the anti-PDL1 antibody (STI-3031, Sorrento Inc., San Diego, CA, USA) with the nanoparticles at various concentrations of antibody. It is important to note that atezolizumab and STI-3031 have similar K_D_, 6.4 × 10^−11^ M and 1.8 × 10^−11^ M, respectively, as assessed by surface plasmon resonance (SPR) (Supplemental Figure S1). The zeta potentials were measured by a zeta-sizer (Malvern Instruments, Malvern, UK), and zeta potential changes were dependent on the concentration of antibody used. The zeta potential of nab-PTX nanoparticles is −29.7 ± 0.404 mV. The zeta potential of the nab-PTX NIC made with 2 mg/mL of antibody increased to −17.2 ± 0.458 mV, while the nab-PTX NIC made with 4 mg/mL increased to −9.1 ± 0.078 mV (Figure 1e) The zeta potential of the nab-SN38 was −20.8 ± 0.153 mV. The zeta potential of the nab-SN38 NIC made with 2 mg/mL of antibody increased to −13.9 ± 0.200 mV, and the NIC made with 4 mg/mL of antibody was −5.8 ± 0.217 mV (Figure 1e) Increasing the antibody concentrations beyond 4 mg/mL had little additional effect on the zeta potential (Figure 1e). This is consistent with our prior results for bevacizumab and rituximab PTX NIC that were tested in phase I trials [39,40].

**Figure 2:**
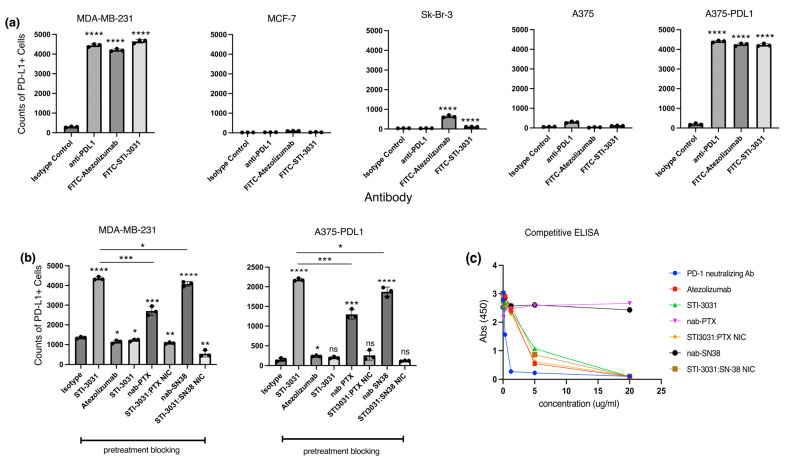
Antibody–Ligand Binding. (**a**) Cell line expression of PD-L1 was determined by flow cytometry using FITC anti-PDL1, alexa fluor 488-conjugated atezoluzumab, and STI-3031. Data are represented in counts of PD-L1-positive cells in triplicate, and the *p*-value was determined with non-parametric two-tailed Student’s *t*-test comparing cells stained with antibody relative to isotype control. (**b**) PDL1-positive cell lines (MDA-MB-231 (**left panel**) and A375-PDL1 (**right panel**)) were utilized to measure the ability of antibody only, nab-PTX, STI3031:PTX NIC, nab-SN38, and STI3031:SN38 NIC to bind cell-bound PD-L1 prior to staining with alexa fluor-conjugated ST-3031. Cells were run by flow cytometry, and data are represented as counts of PDL1+ cells. PD-L1 isotype control and STI-3031 only were utilized as negative and positive controls, respectively; *p*-values were determined using non-parametric Student’s *t*-test comparing cells stained with antibody relative to isotype control or conjugated STI-3031 only. (**c**) Competitive ELISA was employed to confirm antibodies, nab-PTX, STI3031:PTX NIC, nab-SN38, and STI3031:SN38 NIC to inhibit PD-1/PDL1 blocking. Buffer only and PD-1-neutralizing antibodies were used as negative and positive controls, respectively. Curves were compared by two-way ANOVA, and curves were found to be significantly different (*p* = <0.0001). (ns) not significant, (*) *p* = </= to 0.05, (**) *p* = </= to 0.01, (***) *p* = </= 0.001, (****) *p* = </= to 0.0001.

PD-L1 Expression and Ligand Binding: Three breast cancer lines (MDA-MB-231, MCF-7, and Sk-Br-3) and one melanoma line (A375 and PD-L1-transfected A375) were tested for PD-L1 positivity. In addition, an anti-PD-L1 (clone MIH1) flow antibody was compared with alexa fluor 488-conjugated atezolizumab and STI-3031 (Figure 2a). MDA-MB-231 and A375-PDL1 cells were all PD-L1 positive, with anti-PD-L1, atezolizumab, and STI-30331 staining having significantly more PDL1+ cells compared to the isotype control (*p*-value ≤ 0.0001). MCF-7 and A375 WT were PD-L1 negative (Figure 2a). SK-Br-3 cells had no PD-L1+ cells when stained with the positive control anti-PD-L1 while showing some positivity when stained with atezolizumab and STI-3031 with a *p*-value of <0.0001 when compared to the isotype control (Figure 2a).

To confirm that the ligand binding ability of STI-3031 in the context of the STI-3031:PTX NIC and STI-3031:SN38 NIC is retained, a competitive binding experiment was performed by flow cytometry with the PD-L1+ cell lines, MDA-MB-231 and A375-PDL1 cells. Isotype control and STI-3031 labeling were used as negative and positive controls, respectively (Figure 2b). The cell lines were also pretreated with STI-3031, atezolizumab, nab-PTX, STI-3031:PTX NIC, nab-SN38, and STI-3031:SN38 NIC. After pretreatment, the cells were subsequently labeled with alexa fluor 488-conjugated STI-3031. For the MDA-MB-231 cell line, complete blocking was seen with atezolizumab, STI-3031, STI-3031:PTX NIC, and STI-3031:SN38 NIC with *p*-values of 0.016, 0.04, 0.0014, and 0.0012, respectively, which is significantly lower PDL1 expression than the isotype control. Significantly more PDL1 expression was seen when blocking with nab-PTX (*p* = 0.0006) and nab-SN38 (*p* ≤ 0.0001) than the isotype control. The number of PDL1-positive cells was significantly lower than the STI-3031 positive control staining alone in the nab-PTX (*p* = 0.003) and nab-SN38 (*p* = 0.032) blocking, suggesting that the nanoparticles alone block some subsequent staining with STI-3031, possibly due to steric hinderance by the particles. The results with the A375-PDL1 cell line were similar to the results of the MDA-MB-231 cell line, although the total number of PD-L1+ cells were two-fold higher (2178 vs. 4354) than the A375-PDL1 cells. The difference between the number of PD-L1+ cells after blocking with STI-3031, nab-PTX, and nab-SN38 was not significant compared to the isotype control. However, blocking with atezolizumab was significantly different from the isotype control (*p* = 0.03). Blocking with nab-PTX (*p* = 0.0001) and nab-SN38 (*p* ≤ 0.0001) partially blocked subsequent staining with STI-3031, and the number of PDL1+ cells was significantly higher than in the isotype control. Additionally, blocking with nab-PTX (*p* = 0.0003) and nab-SN38 (*p* = 0.014) resulted in significantly fewer PD-L1+ cells than staining with the STI-3031 positive control, like MDA-MB-231 cells. Histograms for this flow experiment are presented in supplemental Figure S2.

Finally, to confirm the ability of atezolizumab, STI-3031, STI3031:PTX NIC, and STI-3031:SN38 NIC to bind PD-L1, a competitive binding ELISA was performed (Figure 2c). In this assay, PD-1 recombinant protein was bound to the plate and recombinant PDL1 protein alone (negative control) or co-incubated with PD-1 neutralizing antibody (positive control), atezolizumab, STI-3031, nab-PTX, STI-3031:PTX NIC, nab-SN38, and STI-3031:SN38 NIC with antibody concentrations ranging from 0 to 20 µg/mL. Atezolizumab, STI-3031, STI-3031:PTX NIC, and STI-3031:SN38 NIC prevented binding of recombinant PDL1 to recombinant PD-1 in a dose-dependent fashion, while nab-PTX and nab-SN38 did not prevent PD1/PDL1 binding. The data are shown at the absorbance of 450 nM, and two-way ANOVA showed a significant difference in the dose–response curves (*p* ≤ 0.0001).

**Figure 3:**
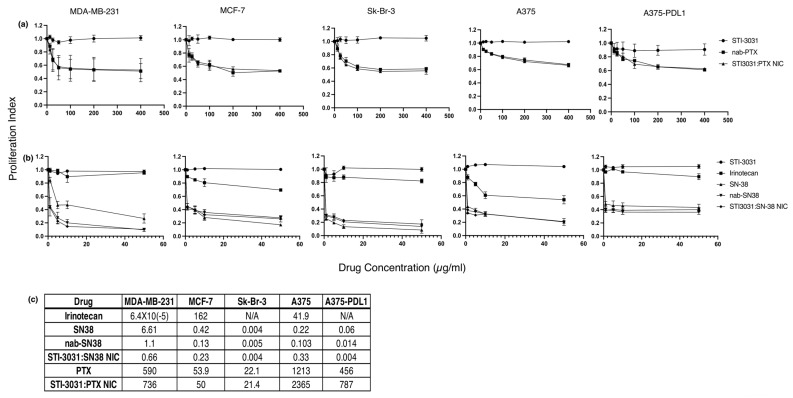
Toxicity of PTX and SN-38. MDA-MB-231, MCF-7, Sk-Br-3, A375 WT, and A375-PDL1 cells were utilized to determine toxicity of STI-301, nab-PTX, and STI3031:PTX NIC (**a**) and irinotecan, free SN-38, nab-SN38, and STI3031:SN38 NIC (**b**). Cells were exposed to drugs overnight, and proliferation was measured using DNA-bound EdU. Two-way ANOVA was utilized to determine the significance of curves in treatment groups for each cell type. Curves were significantly different (*p* = </= 0.00001). (**c**) IC50 values were calculated using non-linear regression in GraphPad Prism (version 10.4.1) and expressed in µg/mL.

Chemotherapy Drug Toxicity: To test the toxicity of the chemotherapy drugs, PTX and SN-38, both alone and in the context of the nanoparticles, and STI-3031-bound NIC, MDA-MB-231, MCF-7, Sk-Br-3, A375, and A375 PDL1 cell lines were utilized. The cells were exposed to antibody alone (STI-3031), nab-PTX, STI3031:PTX NIC (Figure 3a), irinotecan, free SN-38, nab-SN38, or STI3031:SN38 NIC (Figure 3b). The cells were treated with the drug overnight, and proliferation was assessed by the amount of EdU bound to DNA. STI-3031 alone did not impact proliferation in any of the cell lines tested with the range of EdU+ cells ranging from 91 to 100% of the maximal proliferation in untreated cells. The breast cancer lines showed equivalent reduction of proliferation when treated with both nab-PTX and STI3031:PTX NIC. MDA-MB-231 IC50 were 590 and 736 µg/mL, MCF-7 IC50 were 53.9 and 50 µg/mL, and Sk-Br-3 IC50 were 22.1 and 21.4 µg/mL for nab-PTX and STI3031:PTX NIC, respectively. On the other hand, the melanoma lines, A375 WT and A375-PDL1, were somewhat less sensitive to PTX and STI3031:PTX NIC with an IC50 (1231 and 2365 µg/mL) and (456 and 787 µg/mL), respectively. For testing the toxicity of SN-38 nanoparticles and NIC, free SN-38 and its parent drug, irinotecan, were added as controls. Irinotecan had limited toxicity in MDA-MB-231, Sk-Br-3, and A375-PDL1 cells with an IC50 of 6.4 × 10^−5^ for MDA-MB-231 cells, while the IC50 for Sk-BR-3 and A375-PDL1 could not be calculated for lack of a dose response. MCF-7 and A375 WT cells were mildly sensitive to irinotecan with an IC50 of 162 and 41.9 µg/mL. All the cell lines tested were sensitive to free-SN-38, nab-SN38, and STI3031:SN38 NIC with IC50 for MDA-MB-231 (6.61, 1.1, and 0.66 µg/mL), MCF-7 (0.42., 0.13, and 0.23 µg/mL), Sk-Br-3 (0.004, 0.005, and 0.004 µg/mL), A375 WT (0.22, 0.103, and 0.33 µg/mL), and A375-PDL1 (0.06, 0.014, and 0.004 µg/mL). IC50 values are presented in Figure 3c and expressed in µg/mL. Interestingly, whether the cells were PD-L1+ (MDA-MB-231 and A375-PDL1), slightly positive (Sk-Br-3), or negative (MCF-7 and A375) did not significantly affect the level of toxicity of the chemotherapy drugs, possibly due to the long exposure to the drugs.

**Figure 4:**
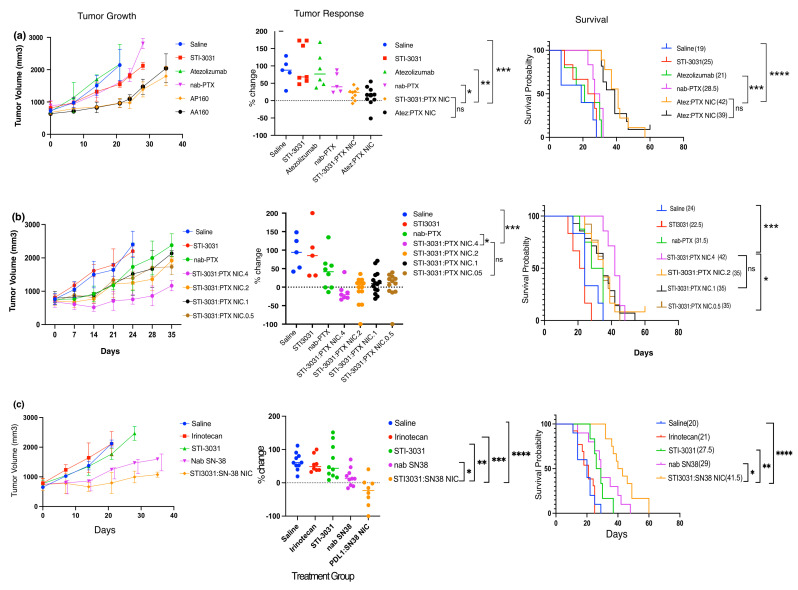
In vivo testing of nab-PTX and nab-SN38 nanoparticles and PD-L1 antibody-coated NIC. First, nab-PTX NIC made with atezolizumab (atez:PTX NIC) was compared to NIC made with STI-3031 (STI3031:PTX NIC). (**a**) Tumor growth (**left** panel), tumor response (**middle panel**), and survival curves are shown (**right panel**). (**b**) The second in vivo experiment compared PD-L1-coated nab-PTX NIC made with 4 concentrations of antibody, 0.5 (STI-3031:PTX NIC 0.05), 1 (STI-3031:PTX NIC.1), 2 (STI-3031:PTX NIC.2), and 4 (STI-3031:PTX NIC.4) mg/mL. Tumor growth (**left panel**), tumor response (**middle panel**), and survival curves (**right panel**) are shown. (**c**) In the final in vivo experiment, PD-L1-coated nab-SN38 NIC was compared to nab-SN38, irinotecan, and antibody only. Tumor growth (**left** panel), tumor response (**middle panel**), and survival curves (**right panel**) are shown. Tumor volume was determined by (length × width2)/2. The line graphs represent the average tumor volumes from 5 mice/group. Tumor response is shown as % change from baseline = [((Volume Day 0) − (Volume day 14 or 21))/Volume Day 0] × 100. For nab-PTX, tumor response was calculated on day 14, and the nab-SN38 tumor responses were calculated on day 21. Kaplan–Meyer curves were generated to show survival. To determine the significance for tumor growth, a 2-way ANOVA was used (*p* = <0.0001). For tumor response, NIC made with 4 mg/mL were compared to each other group individually with non-parametric two-tailed student’s *t*-test. Mantel–Cox log-rank test was utilized to determine median survival significance between NIC made with 4 mg/mL to each other group individually. All statistics were performed in GraphPad Prism software; *p*-values are less than 0.0001 (****), less than 0.001 (***), less than 0.01 (**), and less than 0.05 (*), not significant (ns). Each in vivo experiment was performed 2 times for confirmation of results.

In vivo tumor efficacy: In the first in vivo experiment, nab-PTX was tested against NIC made with STI-3031 or atezolizumab, a clinically approved anti-PD-L1 antibody (Figure 4a). Tumor growth was inhibited most in the NIC groups whether the NIC was made with STI3031 or atezolizumab, as compared to antibody or nab-PTX alone (Figure 4a, left panel). The percent change in tumor size was also calculated (Figure 4a, middle panel). The significance of the tumor size of mice treated with NIC made with STI-3031 was compared to all other individual treatment groups. The difference between the NIC and saline (*p* = 0.0005) or STI-3031 (*p* = 0.0008) was highly significant, while the difference between STI3031:PTX NIC and atezolizumab–PTX NIC was slightly less significant (*p* = 0.0019). The difference between PTX NIC and nab-PTX was also significant (*p* = 0.025), while the difference between the two NICs was not significant (*p* = 0.578), suggesting that antibody targeting with STI-3031 is equivalent to clinically approved atezolizumab. Likewise, survival (Figure 4a, right panel) between the PTX NIC (42 days) and saline (19 days) or antibodies (25 days for STI-3031 and 21 days for atezolizumab) were highly significant (*p* ≤ 0.0001). Additionally, the survival between nab-PTX (28.5 days) and PTX NIC was also significant (*p* = 0.0003), but the difference in survival between the two NIC groups (42 and 39 days) was not significant (*p* = 0.839).

In the second in vivo experiment, PTX NIC were prepared with 0.5, 1, 2, and 4 mg/mL (Figure 4b) to determine if less antibody would confer similar results in tumor response and survival. The NIC made with lower antibody concentrations slowed tumor growth compared to saline, antibody, or nab-PTX alone (Figure 4b, left panel); however, tumor growth was inhibited most by the PTX NIC made with 4 mg/mL STI-3031. Tumor response (Figure 4b, middle panel) determined by percent change from baseline for the NIC made with 4 mg/mL STI-3031 was highly significantly different when compared to saline (*p* = 0.0004) and antibody alone (*p* = 0.0042) and less significant when compared to nab-PTX (*p* = 0.02) like the prior in vivo experiment. Interestingly, the tumor response between the NIC made with any concentration of antibody was not significant at day 14. The tumor response between NIC made with any concentration of antibody was significantly different for saline (*p* = </=0.0007) and antibody only (*p* = 0.004); however, only the NIC made with 2 mg/mL of antibody was significant from nab-PTX (*p* = 0.016). The NIC made with 1 mg/mL (*p* = 0.104) and 0.5 mg/mL (*p* = 0.067) were not significantly different from nab-PTX. Survival between the NIC made with 4 mg/mL of antibody and those made with 1 mg/mL (*p* = 0.182) and 2 mg/mL (*p* = 0.148) of antibody were not significant (42 and 35 days, respectively), however, the difference between 4 mg/mL and 0.5 mg/mL was significant (*p* = 0.043). The difference in survival (Figure 4b, right panel) between the NIC made with 4 mg/mL was highly significant when compared to saline (*p* = 0.0005), antibody alone (*p* = 0.0002), and nab-PTX (*p* = 0.001).

In the final in vivo experiment, nanoparticles made with SN-38 were tested against STI3031:SN38 NIC (Figure 4c). Tumor growth was inhibited by both the nab-SN38 and the NIC made with STI-3031 when compared to saline, irinotecan, and STI-3031 alone (Figure 4c, left panel). The day 21 tumor response (Figure 4c, middle panel) between STI3031:SN38 NIC was significantly different from all other groups: saline (*p* = 0.0009), STI3031 (*p* = 0.006), irinotecan (*p* = <0.0001), and nab-SN38 (*p* = 0.0312). Notably, the tumor response at day 14 between STI3031:SN-38 NIC and nab-SN-38 was not significant (*p* = 0.81), while SN-38 NIC compared to the other groups was significant at day 14. Similarly, the median survival (Figure 4c, right panel) between the NIC and all other groups was significant: saline and irinotecan (*p* = <0.0001), STI-3031 (*p* = 0.02), and nab-SN38 (*p* = 0.03).

## 3. Discussion

The classic breast cancer tumor antigen, Her-2/neu, is currently the topic of ADC targeting and has for years been the target of therapeutic monoclonal antibodies. In the setting of TNBC, this target is not available. Herein, we postulated that an immunologically relevant molecule, PDL1 expressed by cells of TNBC, could potentially serve as a target of an ADC approach [1,2]. Some of the most recent developments in the treatment of TNBC include nanoparticle formulations of PTX, irinotecan, and SN-38 in combination with ICI, particularly anti-PD-L1 blocking drugs [21,34]. To provide other treatment options for these patients, we developed a PDL1-targeted nano-immune conjugate that consists of albumin-bound PTX or SN38 nanoparticles coated with an anti-PD-L1 antibody. Here, we demonstrate that these albumin nanoparticles can be reproducibly manufactured and bound with an antibody (atezolizumab and STI-3031). We also show that the chemotherapy drug and antibody retain their individual functions in the context of the NIC. Furthermore, in in vivo testing, the NIC improves tumor response and median survival in a xenotransplant model of TNBC.

Currently, there are nearly 300 clinical trials testing ICI alone or in combination with chemotherapy in breast cancer, suggesting that these types of treatments are of great interest [3]. Until recently, breast cancer has been viewed as a relatively immunologically inert tumor, which would make it less amenable to ICI [47]. In addition, since TNBC is hormone receptor negative, treatment options are even more limited. However, Adams et al. analyzed data from two phase III trials of TNBC and found that tumor infiltrating T-cells (TIL) were a positive prognostic indicator affecting disease-free and overall survival, suggesting the possibility that ICI could be a viable treatment option for TNBC [48]. Furthermore, studies have shown that up to 32% of primary TNBC tumors are PD-L1 positive, partially depending on the stage of disease [49]. As such, atezolizumab given with nab-PTX was FDA-approved for the treatment of TNBC in 2019 [33]. Additionally, an ADC containing SN-38 covalently linked to anti-TROP2, IMMU-132, has also been FDA approved for relapsed and refractory TNBC [9]; therefore, our NIC containing PTX or SN-38 bound to anti-PD-L1 is a logical option for TNBC treatment as suggested by our in vivo results (Figure 4).

Another interesting aspect of the NIC containing PTX or SN-38 and targeted by anti-PDL1 is the fact that the chemotherapy drugs, as well as the antibody, are immunomodulatory. The fact that PD-L1 expressed in the tumor is immunosuppressive and is a negative prognostic indicator for several cancer types is well established [50]. PD-L1 is expressed on host immune cells as well as tumor cells, which has been shown to result in T-cell apoptosis [51,52]. Treatment with anti-PDL1 therapy results in the enhancement of antigen presentation by dendritic cells (DCs) to help generate an effective CD8+ T-cell response and effects tumor-associated macrophage activity [52]. Studies have shown that PTX impacts immunity in several ways. For example, PTX can reactivate innate immunity, reprogram tumor-associated macrophages from immunosuppressive M2 to immune active M1, and enhance antigen presentation of tumor antigens, which can elicit a productive anti-tumor response by CD8+ T-cells [53]. SN-38 has also been proven to enhance anti-tumor immunity by increasing interferon-gamma expression by natural killer (NK) cells and promoting NK and T-cell infiltration in mouse tumor models of head and neck squamous cell carcinoma [54]. In our current studies, the anti-PDL1 serves as a tumor targeting agent only because we have only tested the anti-PD-L1 NIC for tumor response in athymic nude mice; hence, we cannot access immune system fluctuations caused by treatment. Therefore, we are currently developing a syngeneic mouse tumor model so we can assess the changes in immunity systemically and in the tumor microenvironment to understand how immune changes affect tumor response beyond that of just the cytotoxicity of the chemotherapy.

Developing the ability to target chemotherapy to the tumor and away from normal tissues has been long sought after. Despite years of research, the number of antibody-targeted chemotherapy drugs is still limited, with a few notable exceptions [55,56]. The benefit of our nano-immune conjugate relative to other ADCs is that safe, naturally occurring albumin provides the link between the antibody and the chemotherapy by non-covalent bonds [39,40]. As a result, these NICs do not require cellular uptake and enzymatic cleavage of a linker for the cytotoxic agent to affect tumor growth. The only requirement for these NICs is to get to the tumor microenvironment, where they naturally disassociate, allowing the chemotherapy to have its cytotoxic effect. Like the NIC we have previously described, these anti-PDL1-coated PTX and SN-38 nanoparticles can be reproducibly manufactured. We have also shown that the individual drugs retain their function in the context of the NIC. Additionally, these NICs show efficacy in a xenotransplant model of human TNBC, including increased tumor response and median survival in the NIC groups relative to the single drugs alone.

## 4. Materials and Methods

Nanoparticle Manufacturing and Testing: The schema for nanoparticle manufacturing with PTX and SN-38 is shown in Figure 1a. PTX and SN-38 were purchased from Selleck Chemicals (Houston, TX, USA). The size of the nanoparticles was measured in saline, and the zeta potential was measured in HPLC-grade water, both at a concentration of 100 µg/mL of drug using the Zeta-sizer (Malvern Panalytical, Malvern, UK). For the stability assay, the nab-PTX and nab-SN38 were diluted to various drug concentrations in saline or simulated plasma (5% human serum albumin in water), and their particle sizes were measured by a Zeta-sizer (Malvern Panalytical, Malvern, UK).

Recombinant human PD-L1 protein (Abcam, Cambridge, United Kingdom (UK)) was immobilized onto a CM5 sensor chip via standard amine coupling using the Biacore X100 system (Cytiva, Marlborough, MA, USA). The immobilization was performed in 10 mM sodium acetate buffer (pH 5.5) to achieve an immobilization level of 25.9 RU. ST3031 and atezolizumab were prepared in HBS-EP+ buffer (10 mM HEPES, 150 mM NaCl, 3 mM EDTA, 0.05% *v*/*v* surfactant P20, pH7.4) at concentrations of 0.008 µg/mL, 0.04 µg/mL, 0.2 µg/mL, 1.0 µg/mL, 5.0 µg/mL, and 25.0 µg/mL, respectively. The kinetics were measured using multi-cycle kinetics at a flow rate of 30 µL/min. The association phase was set to 180 s, followed by a dissociation phase of 18,000 s. The chip surface was regenerated using 50 mM NaOH. Kinetic parameters (ka, kd, KD) were determined using Biacore X100 Evaluation Software (version 2.0.1) with the “Bivalent Analyte” fitting mode. The quality of the fit was evaluated based on Chi^2^ values and residual plots.

Formation of nano-immune conjugate: To create nano-immune conjugates of the PTX and SN38 nanoparticles (nab-PTX and nab-SN38), the nanoparticles were co-incubated with 4 mg/mL of anti-PD-L1 at room temperature for 30 min with gentle shaking. Clinical grade atezolizumab was purchased from the Mayo Clinic Pharmacy, and STI-3031 was obtained from Sorrento Therapeutics (San Diego, CA, USA). All NICs for in vitro and in vivo testing were made using an antibody at 4 mg/mL except for measuring zeta potential, where concentrations of 0–8 mg/mL were used. Also, NIC was made at various concentrations of STI-3031 for antibody dose–response in one in vivo experiment.

Cell Culture: MDA-MB-231, MCF-7, Sk-Br-3, and A375 cells were purchased from ATCC. The cells were thawed and cultured in DMEM with 10% FBS (Gibco, Billings, MT, USA) and 1% penicillin, streptomycin, and glutamine (Gibco, Billings, MT, USA). The cells were expanded and frozen. For all mouse experiments, the cells were expanded in less than 10 passages and implanted into mice. MDA-MB-231, MCF-7, Sk-Br-3, and A375 cells were also used for all in vitro assays. The cells were routinely tested for PD-L1 expression with PE-conjugated anti-human PD-L1 (Clone MIH1, BD Bioscience, Franklin Lakes, NJ, USA).

PD-L1 expression: PD-L1 expression was enumerated in MDA-MB-231, MCF-7, Sk-Br-3, A375 WT, and A375-PDL1 cells. Atezolizumab and STI-3031 were purified with protein G columns as per manufacturer’s instructions (Thermofisher, Waltham, MA, USA). Briefly, columns were washed with 2× with water, and 0.5 mg of antibody was run over the column. After column washing with PBS, the antibody was eluted from the column with 0.1 M glycine-HCl at pH 3.0. The antibody fractions were neutralized with 1 M sodium bicarbonate at pH 11.5. The purified protein concentration was measured at wavelength 280 by NanoDrop (Molecular Devices, San Jose, CA, USA). The antibodies were then directly conjugated to Alexa Fluor 488 or Alexa Fluor 647 using a labeling kit (Thermofisher, Waltham, MA, USA). Antibodies were diluted to 0.5 mg/mL and incubated in 1 M sodium bicarbonate and fluorochrome for 1 h at room temperature. Sepharose columns were utilized to remove free fluorochrome. A single cell suspension of tumor cells was created using Miltenyi Gentle MACS (Miltenyi Biotech, Bergisch, Germany) and M-tubes with the preprogrammed protocol for single cell suspensions. Cultured cells were harvested using trypsin–EDTA. Labeled cells were run by flow cytometry and analyzed by GuavaSoft version 3.1.1 (Guava 8HT, Millipore Sigma, Burlington, MA, USA).

For staining, cultured MDA-MB-231, MCF-7, Sk-Br-3, and A375 cells were incubated with 1 ng/mL of labeled antibody in FACS buffer (0.5% BSA and 0.5 M sodium azide in PBS).

In vitro toxicity: To determine the toxicity of the PTX and SN38 in the nanoparticles and NIC, the Click-iT Edu kit (ThermoFisher, Waltham, MA, USA) was utilized to determine cell toxicity by enumerating the percentage of cells still proliferating after treatment. Briefly, assays were performed by plating 7.5 × 10^5^ cells in each well of a 24-well plate. The cells were treated with the anti-PDL1 antibody only, STI3031 (Sorrento, San Diego, CA, USA), nab-PTX, and PTX NIC at concentrations from 12.5 to 400 mg/mL or irinotecan (Selleck Chemicals, Houston, TX, USA), free SN-38 (Selleck Chemicals, Houston, TX, USA), nab-SN38, and SN38 NIC at concentrations between 0 and 50 mg/mL of chemotherapy and 5–160 ng/mL of PD-L1 antibody overnight. Ten mM EdU was added to all treatment wells and positive control wells with no treatment. The negative control was untreated cells with no EdU and fluorescent label. The next day, the cells were harvested, washed, fixed, and permeabilized with 10% saponin. Following permeabilization, Alexa fluor 647 azide was added to the cells for 30 min in labeling buffer to detect DNA-incorporated EdU. Cells were immediately run by flow cytometry (Guava 8HT, Millipore Sigma, Burlington, MA, USA). The number of positive, proliferating cells was determined (GuavaSoft, Millipore Sigma, Burlington, MA, USA), and the proliferation index was calculated by dividing the proliferation percentage of treated cells by the percentage of proliferating cells in the untreated well. 

Ligand Binding: To determine ligand binding of the antibody, the nanoparticles and NIC were prepared as described above. After formation, the NIC was spun at 6000 rpm for 10 min to remove any unbound antibodies. After removing unbound antibodies, 1 × 10^5^ MDA-MB-231 and A375-PDL1 cells were incubated with antibody only, nab-PTX, nab-SN38, or the PD-L1 NIC for 30 min at room temperature. After incubation, the cells were washed 2× and incubated with alexa fluor 488-conjugated STI-3031 for 30 min. After the final washes, themin cells were analyzed by flow cytometry to determine whether the cold antibody, nab-PTX, nab-SN38, or STI-3031-coated NIC effectively blocked subsequent staining with fluorescently labeled STI-3031. Labeled cells were run immediately by flow cytometry (Guava 8HT, Millipore Sigma, Burlington, MA, USA). Histogram plots were generated (GuavaSoft, Millipore Sigma, Burlington, MA, USA) comparing isotype labeled negative control, positive controls labeled with Cy5 conjugated STI-3031, and cells pretreated with antibody only, nab-PTX, nab-SN38, and the two NICs. To confirm ligand binding results, a competitive ELISA (Acro Biosystems Newark, Newark, DE, USA) was utilized. Atezolizumab, STI-3031, nab-PTX, STI-3031:PTX NIC, nab-SN38, and STI3031:SN38 NIC were co-incubated at concentrations from 0 to 20 µg/mL with recombinant PD-L1 protein to wells of a 96-well plate in which recombinant PD-1 was bound. A PD-1 neutralizing antibody was used as a positive control, and buffer only was the negative control. Samples were incubated for 90 min at 37 °C. Wells were then washed in wash buffer as stated by the manufacturer’s protocol. After washing 3 times, biotin-conjugated PD-1 protein was added to the wells and incubated for 1 h at 37 °C. The washes were repeated, streptavidin–HRP was added, and the plate was incubated for 20 min at 37 °C. Finally, the signal was developed with substrate, and development was stopped with 2N HCl. Absorbance was measured at 450 nM with an ELISA plate reader (Molecular Devices, San Jose, CA, USA).

In vivo efficacy: Mouse experiments were approved by and completed in accordance with Mayo Clinic IACUC under the protocol number A00005883-21-R24 and were approved on April 22, 2021. To test the in vivo efficacy of the PD-L1-targeted PTX and SN-38 nanoparticles, 5 × 10^6^ cultured MDA-MB-231 cells were injected into the right flank of female athymic nude mice (Envigo, Indianapolis, IN, USA). Mice were allowed to acclimate for 7 days before treatment and were approximately 7 weeks old. Tumors were implanted in 10–15 mice/group because not all mice will have engrafted tumors. Treated groups had at least 8 mice/group. When tumors had reached approximately 750 mm^3^, mice were divided into treatment groups: saline, STI-3031 only, irinotecan, nab-PTX, nab-SN38, STI-3031 coated nab-PTX, and nab-SN38. Mice were treated with 18 mg/kg of antibody, 15 mg/kg SN-38, and 45 mg/kg PTX, whether alone or in nanoparticle and NIC formulation. Mouse tumors were monitored 3 times/week. Tumor volume was calculated by the formula: (length × width2)/2. Tumor response (% change) was calculated by the formula: {(tumor size (day14 of day 21) − tumor size (day0))/tumor size (day0)} × 100. Kaplan–Meier curves were generated for median survival. All data were graphed and statistically evaluated in GraphPad Prism (La Jolla, CA, USA). To determine statistical significance between groups for tumor response, a two-tailed student’s *t*-test was utilized, and the Mantel–Cox Log-rank test was utilized to determine the statistical significance of survival. The various NICs made with 4 mg/mL of antibody were compared to all other groups individually to determine significance.

Statistical Analysis: To compare PD-L1 expression, cellular ligand binding, and tumor response, a non-parametric two-tailed Student’s *t*-test was utilized. In the flow cytometry for PD-L1 expression, antibody staining was compared to isotype control. In the cellular ligand binding assay, PD-L1 expression for each treatment was compared to the isotype control and STI-3031 staining without blocking treatment. In the in vivo tumor response experiments, treatment groups were compared individually to saline treatment. For the toxicity assays, competitive ELISA, and tumor growth curves, the 2-way ANOVA test was utilized. For in vivo survival, the Mantel–Cox log-rank test was utilized to determine the significance of the treatment groups to saline. To determine the number of mice needed/group, power analysis was performed for 90% confidence. Elimination of mice when necessary was due to lack of tumor engraftment.

## 5. Conclusions

Over the last decade, the melanoma research program at Mayo Clinic has spent significant time developing a drug delivery platform. This drug delivery platform uses human serum albumin as the carrier for both small molecule hydrophobic chemotherapy drugs and clinically relevant IgG1 antibodies. Phase I clinical responses for the bevacizumab and rituximab nanoparticles are very promising, with about 55% and 85% response rates in gynecological malignancies and refractory and relapsed CD20+ non-Hodgkin’s lymphoma, respectively.

Herein, we present data using two anti-PDL1 antibodies, atezolizumab and STI-3031, on nab particles containing PTX and SN38. Importantly, a reproducible method for manufacturing nanoparticles containing SN38 was developed. The manufacturing processes for nab-PTX and nab-SN38 are slightly different due to drug solubility (Figure 1a). The ability to use multiple chemotherapy drugs and antibodies separately or together in the NIC will expand the types of cancer that can be treated.

Finally, because anti-PDL1 is an immunologically relevant antibody, it is important to test these PDL1-targeted nanoparticles in immunocompetent syngeneic mouse models, which is an ongoing topic of research in our laboratory. Furthermore, phase I clinical testing of PDL1-targeted nab-PTX and nab-SN38 will be extremely valuable in understanding the immunological result of including anti-PDL1 in these NICs. The impact of the NIC on tumor immunity remains to be tested. Given that PD-L1 is expressed on many different cancer types [50], these NICs may have broad application in clinical cancer therapy, and clinical testing should be pursued.

## Data Availability

All data supporting this manuscript are presented within.

## Supplementary Materials

The supporting information can be downloaded at https://www.mdpi.com/article/10.3390/ijms26073295/s1.

## Abbreviations

The following abbreviations are used in this manuscript:

Nab	Nanoparticles of Albumin
NIC	Nano-immune conjugate
PTX	paclitaxel
PD-L1	Programmed Cell Death Ligand 1
TNBC	Triple-Negative Breast Cancer
SPR	Surface Plasmon Resonance
ELISA	Enzyme-linked immunsorbant assay
