# Supplementary material for: PD-L1-Targeting Nanoparticles for the Treatment of Triple-Negative Breast Cancer: A Preclinical Model
Source: Int J Mol Sci. 2025 Apr 2;26(7):3295. doi: 10.3390/ijms26073295 (PMC11989481; doi:10.3390/ijms26073295)
Supplement: Supplementary file 1 [file ijms-26-03295-s001.zip › ijms-3529492-supplementary.pdf]

(a)

Analyte (Sample)	Concentration
Atezolizumab	0.008ug/ml
Atezolizumab	0.04ug/ml
Atezolizumab	0.2ug/ml
Atezolizumab	1.0ug/ml
Atezolizumab	5.0ug/ml
Atezolizumab	25.0ug/ml
Atezolizumab	125ug/ml

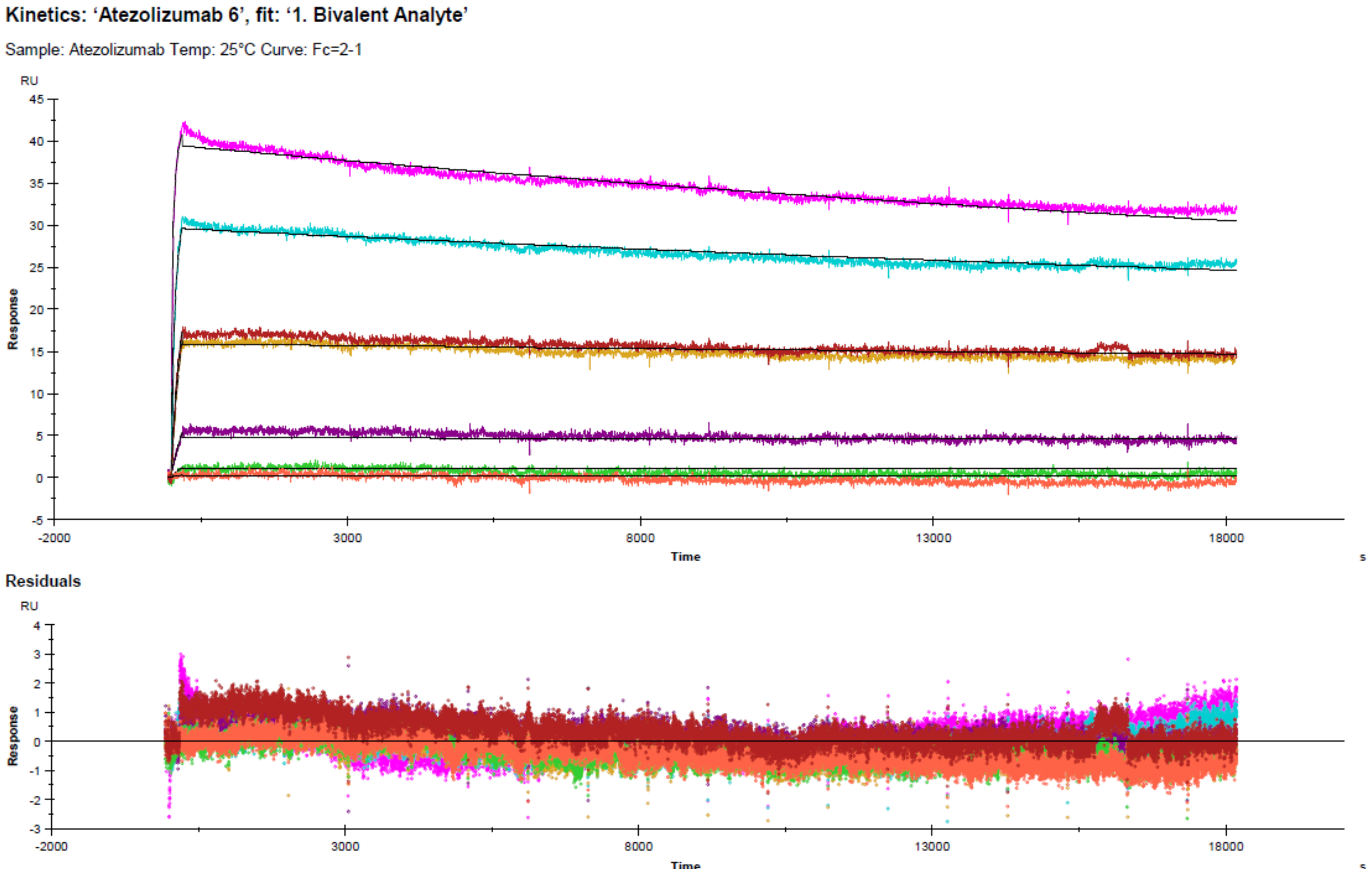

K_D=Kd1/Ka1=1.723E-5/2.697E+5=6.4E-11M=64pM.

Rmax: 41.36RU; Chi^2: 0.322RU^2.

Results: The K_D of Atezolizumab to recombinant human PD-L1 is 6.4E-11M (64pM).

(b)

Analyte (Sample)	Concentration
STI3031	0.008ug/ml
STI3031	0.04ug/ml
STI3031	0.2ug/ml
STI3031	1.0ug/ml
STI3031	5.0ug/ml
STI3031	25.0ug/ml
STI3031	125ug/ml

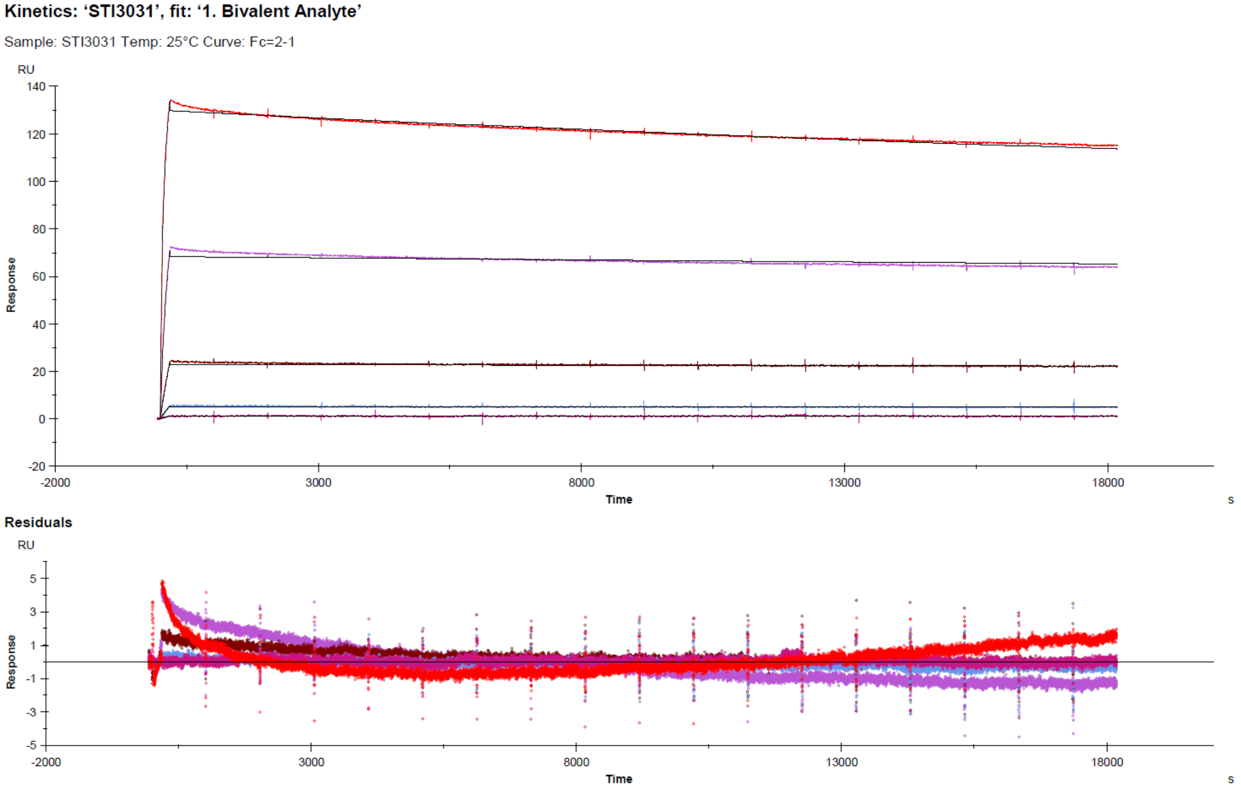

K_D=Kd1/Ka1=1.282-5/6.943E+5=1.8E-11M=18pM.

Rmax: 153.0RU; Chi^2: 0.495RU^2.

Results: The K_D of STI3031 to recombinant human PD-L1 is 1.8E-11M (18pM).

Supplemental Figure S1 Binding kinetics of atezolizumab and STI-3031. The K_d of atezolizumab (a) and STI-3031 (b) were determined by surface plasmon resonance utilizing the Biacore 100. The concentrations of each antibody used in the kinetics assay is shown in the table and binding curves of each antibody are shown. K_d of the antibodies were found to be similar with K_d of 6.4 x 10^{-11} for atezolizumab and 1.8 x 10^{-11} for STI-3031.

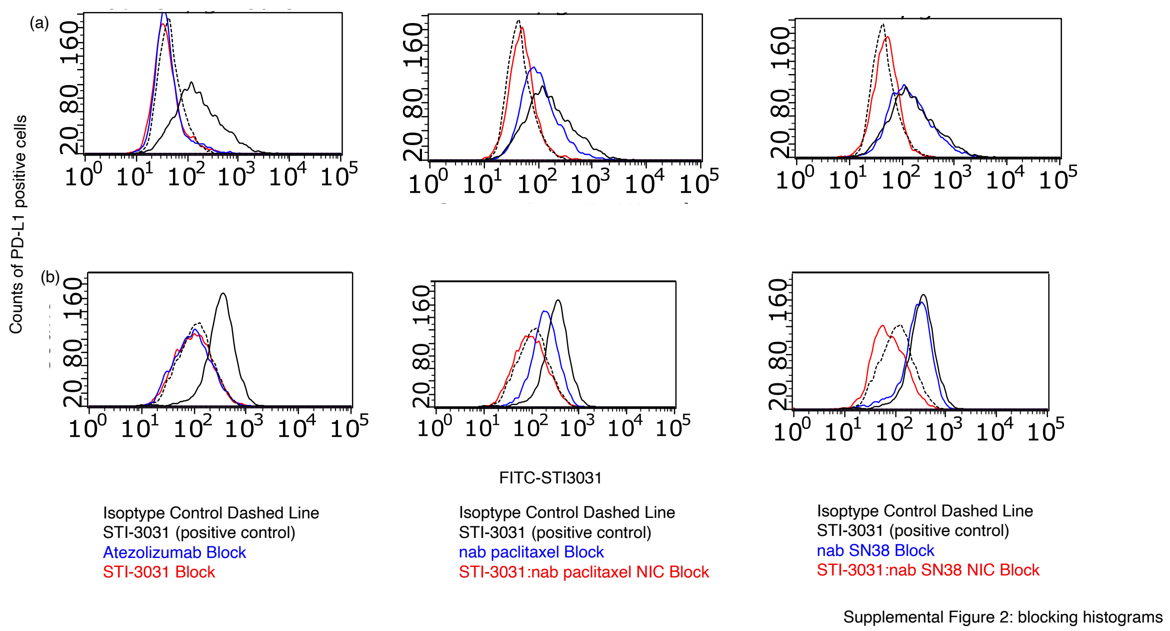

Supplemental Figure S2 Histograms of ligand binding. Example histograms are shown for MDA-MB-213 (a) and A375-PDL1 (b) for cellular ligand bind of STI-3031, atezolizumab, STI-3031, nab-paclitaxel, STI3031:paclitaxel NIC, nab-SN38, and STI3031:SN38 NIC. Isotype control was the negative control and conjugated STI-3031 without pretreatment was used as a positive control. The negative and positive controls are shown in every panel. The left panel in (a) and (b) shows the controls relative to the atezolizumab and STI-3031 pretreatment. The middle panels show the controls relative to the nab-paclitaxel and STI-3031:paclitaxel NIC pretreatment. The right panels show the controls relative to the nab-SN38 and the STI3031:SN38 NIC.
